# Impact of frequent intradialytic hypotension on quality of life in patients undergoing hemodialysis

**DOI:** 10.1186/s12882-023-03263-6

**Published:** 2023-07-14

**Authors:** Jianhua Wang, Jing Yao, Xiaoye Zhu, Tingting Wang, Jianda Lu, Qiubo Wei, Jun Xue, Yuanhao Wu, Li You

**Affiliations:** Department of Nephrology, Huashan Hospital Affiliated to Fudan University, Baoshan Branch, Shanghai, China

**Keywords:** Intradialytic hypotension (IDH), Quality of life (QoL), Predialysis, Blood pressure (BP), Systolic blood pressure (SBP)

## Abstract

**Background:**

Intradialytic hypotension (IDH) is frequently accompanied by symptoms of nausea, dizziness, fatigue, muscle spasm, and arrhythmia, which can adversely impact the daily lives of patients who undergo hemodialysis and may lead to decreased quality of life (QoL). This study employed the KDQOL™-36 scale to evaluate the impact of frequent IDH, based on the definition determined by predialysis blood pressure (BP) and nadir systolic blood pressure (SBP) thresholds, on the QoL of patients.

**Methods:**

This is a single center retrospective cohort study involving 160 hemodialysis patients. We enrolled adult patients with uremia who received routine hemodialysis (4 h/time, 3 times/week) from October 1, 2019, to September 30, 2021. Frequent IDH was defined as an absolute nadir SBP < 90 mmHg occurring in no less than 30% of hemodialysis sessions when predialysis SBP < 159 mmHg (or < 100 mmHg when predialysis BP ≥ 160 mmHg).The differences between patients with and without frequent IDH were compared using the independent t test, Kruskal‒Wallis test, or chi-square test. The primary visit was at month 36, and the remaining visits were exploratory outcomes.

**Results:**

Compared to patients with infrequent IDH at baseline, those with frequent IDH had significantly lower scores on the symptoms and discomfort of kidney disease dimension at all follow-up points (P < 0.05). The symptoms and discomfort of kidney disease dimension were worse in patients with frequent IDH. Those with frequent IDH had a significantly poorer QoL regarding the dimensions of symptoms and discomfort of kidney disease and the impact of kidney disease on life.

**Conclusions:**

The findings of the study suggest an association between frequent IDH and QoL dimensions of symptoms and discomfort of kidney disease and the impact of kidney disease on life dimension under the definition of frequent IDH.

## Introduction

Intradialytic hypotension (IDH) is one of the most common complications during hemodialysis [1]. Its presence not only affects the process of dialysis treatment but is also closely associated with a range of adverse outcomes, including cardiac dysfunction, arteriovenous fistula thrombosis, cerebral ischemia, and residual renal function impairment [2]. However, there is currently no consensus regarding the diagnostic criteria for IDH due to wide heterogeneity in its definition worldwide. The Kidney Disease Outcomes Quality Initiative (K/DOQI) guidelines propose a definition of IDH as a decrease of ≥ 20 mmHg in systolic blood pressure (SBP) or a decrease of ≥ 10 mmHg in mean arterial pressure (MAP), accompanied by hypotensive symptoms such as headache, general fatigue, convulsions, nausea, vomiting, and restlessness during dialysis [3]. Alternatively, a retrospective study has proposed a definition of IDH based on a nadir SBP of less than 90 mmHg for patients with a predialysis SBP of less than 159 mmHg (or less than 100 mmHg for patients with a predialysis SBP of greater than 160 mmHg) [4]. This definition of IDH was identified as being most strongly associated with mortality when its frequency was ≥ 30% [4].

IDH is frequently accompanied by symptoms of dizziness, fatigue, muscle spasm, and arrhythmia, which can adversely impact the daily lives of patients who undergo hemodialysis and may lead to decreased quality of life (QoL) [5, 6]. QoL is a measure that provides a comprehensive assessment of a patient’s real health status and is widely used in clinical practice and decision-making. Timely and effective QoL monitoring of hemodialysis patients can help to better understand the condition, improve clinical management strategies, and ultimately improve their health. However, to date, few studies have addressed the QoL outcomes associated with the particular definition of IDH. Kuipers J et al. found no significant associations between the mental summary score or the physical summary score and the proportion of dialysis sessions that fulfilled the full European Best Practice Guideline definition by using the 36-Item Short-Form Health Survey, but found a significant association between QoL and a simple patient-reported intradialytic symptom score [7].

The Renal Disease-36 Quality of Life (KDQOL™-36) scale is one of the most widely used measures for evaluating QoL among patients with end-stage renal disease (ESRD) [8]. The scale is designed to assess the particular issues that individuals with kidney disease may experience, allowing for the collection of accurate data regarding the various implications of kidney disease on QoL [9].

To better clarify the relationship between IDH and the QoL of patients and stretch a clinically valuable definition of IDH, this study employed the KDQOL™-36 scale to evaluate the impact of frequent IDH on the QoL of patients based on the definition determined by predialysis BP and nadir SBP thresholds.

## Methods

### Patients

This is a single-center retrospective cohort study. We enrolled 163 adult patients with uremia who received routine hemodialysis (4 h/time, 3 times/week) at the hemodialysis center of Huashan Hospital Affiliated to Fudan University, Baoshan Branch, from October 1, 2019, to September 30, 2021. Patients with the following conditions were excluded from the study: (1) Short life expectancy (< 1 year); (2) Potential for short-term renal function restoration or other renal replacement therapies (< 6 months); (3) Organ failure (other than kidney); (4) Diagnosis of an untreated solid or hematological tumor within the past 5 years; (5) Diagnosis of active gastrointestinal bleeding within the past one month; (6) Uncorrected or uncorrectable congestive heart failure; (7) History of myocardial infarction, cerebral infarction, or cerebral hemorrhage in the past three months; (8) Dementia or inability/refusal to measure blood pressure on the upper arm; or (9) Inability to cooperate with the study or refusal to sign an informed consent form. The study was approved by the Medical Ethics Committee of Huashan Hospital Affiliated to Fudan University (IRB No. KY2021-609).

### Study protocol

The blood pressure of all participants in this study was measured before and after dialysis using an upper arm cuff sphygmomanometer. Measurement occurred automatically every 30 min during each dialysis. IDH was defined based on the predialysis BP and nadir SBP thresholds, namely, a nadir SBP of < 90 mmHg (or < 100 mmHg for those with a predialysis BP of ≥ 160 mmHg) [4]. Frequent IDH was defined as an incidence of IDH exceeding 30% within a three-month period. Following study inclusion, participants first underwent a 30-day adaptation period, followed by a three-month (July 1, 2019, to September 30, 2019) exposure assessment period for the determination of baseline frequent IDH. Subsequently, the patients were followed up for 36 months.

During the 27 months of follow-up, patients were asked to complete a paper questionnaire. Most of the patients (148 cases) were able to complete the questionnaire independently, and 12 patients who were unable to care for themselves, partially cared for themselves, or had a low education level completed the questionnaire with the help of nurses. The electronic questionnaire APP was used in the thirtieth month. Most of the patients could complete the questionnaire by themselves, and a few patients could complete it with the help of nurses.

**Fig. 1:**
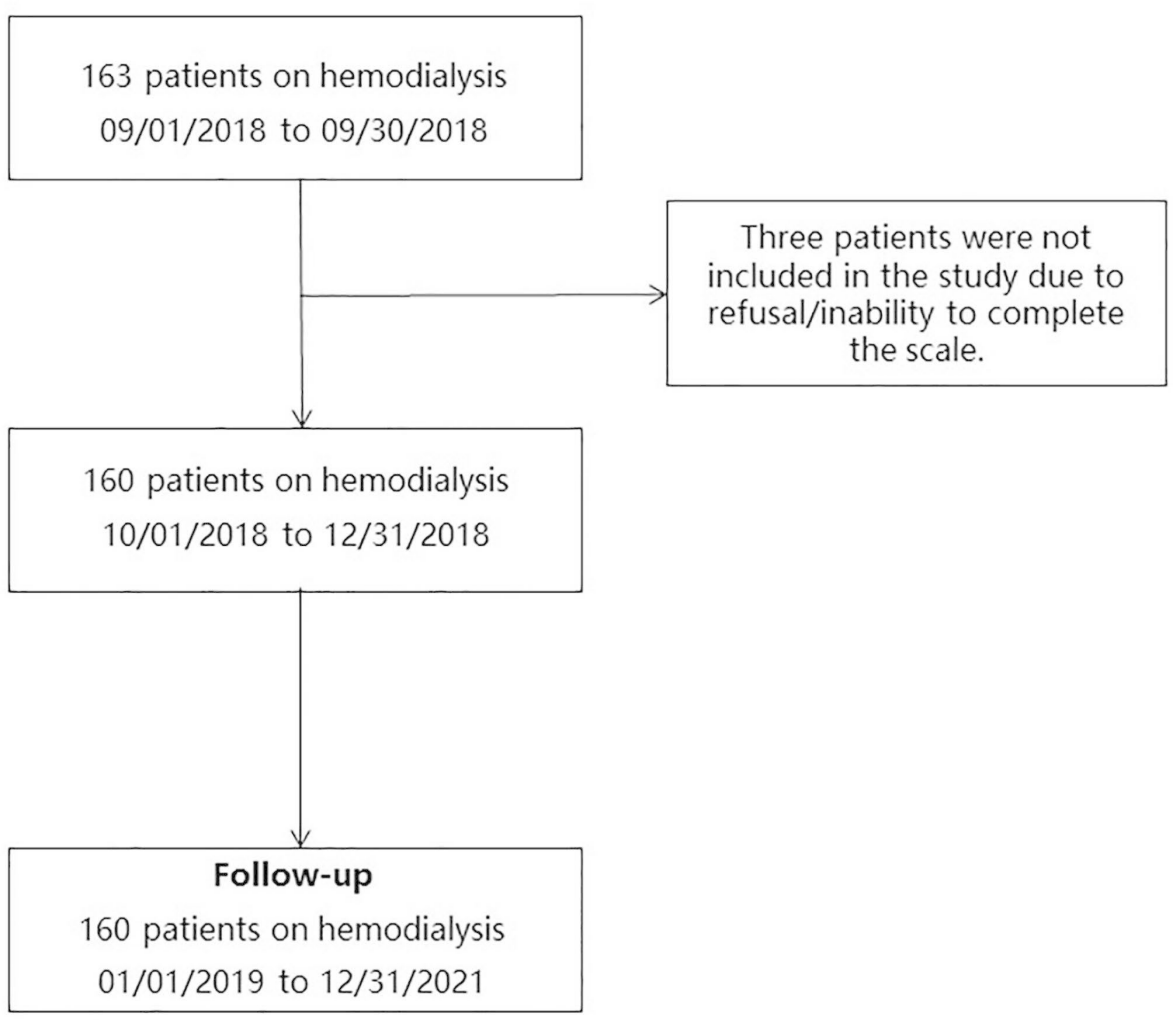
Flowchart of the study population

The following data were collected for each patient: mean interdialytic weight gain, actual ultrafiltration volume, predialysis SBP, QoL, etc. QoL was assessed using the KDQOL™-36 scale [8]. Patients included in this study completed the scale by themselves or with assistance after hemodialysis. Three patients were not included in the study due to refusal/inability to complete the scale. This scale consists of 36 items, which cover five dimensions: symptoms and discomfort of kidney disease, impact of kidney disease for life, burden kidney disease brings on life, physiological health, and mental health. The QoL score was determined according to the scoring criteria provided by the KDQOL™-36 scale. A higher score indicates better QoL. Routine blood and biochemical examinations and the QoL assessment were performed at baseline (three months prior to follow-up) and every three months thereafter (Fig. 1).

### Statistical analysis

The baseline characteristics of patients were descriptively analyzed, and differences between patients with and without frequent IDH were compared using the independent t test, Kruskal‒Wallis test, or chi-square test. The association of QoL and IDH was examined by comparing between-group differences (frequent IDH vs. infrequent IDH) in KDQOL™-36 scores during the follow-up. A mixed-effects model with repeated measurements was used to explore this association, in which the dependent variables were the KDQOL™-36 scores, with confounding adjustments for sex, age, cause of ESRD (using chronic glomerulonephritis as reference), type of VA (using arteriovenous fistula as reference), duration of dialysis, mean interdialysis weight gain, serum creatinine (Scr), hemoglobin (Hb), visit (treated as a random effect), and interaction of IDH group and visits. The effects were measured by the least squares mean difference (LSMD) and its 95% confidence intervals (CIs). The primary outcome was the score on the KDQOL™-36 scale at month 36. Secondary outcomes were the score on the KDQOL™-36 scale at months 3 to 33, the mean between-group difference in QoL scores over time, and the variability of the between-group difference over time. The primary analysis was based on the dataset imputed by the last observation carried forward (LOCF), and a sensitivity analysis without missing data imputation was also conducted. All statistical analyses were performed using SAS 9.4.

## Results

### Patients

A total of 160 patients undergoing maintenance hemodialysis were included. Of these, 102 (63.8%) were male and 58 (36.2%) were female, with a mean age of 62.3 ± 11.3 years and a median duration of hemodialysis of 53 months. The mean interdialytic weight gain during the baseline period was 2.19 ± 0.84 kg. A total of 48 (30%) patients met the definition of frequent IDH. The baseline characteristics are presented in Table 1.

**Table 1:** Baseline description and comparison

Factor	Classification	Frequent IDH		Chi^2^/t	P
		No (n = 112)	Yes (n = 48)		
Gender, n(%)	Male	32 (28.6%)	26 (54.2%)	9.53	**0.0020**
	Female	80 (71.4%)	22 (45.8%)		
ESRD etiology	CGN	31 (27.7%)	15 (31.2%)	8.61	**0.0350**
	DM	23 (20.5%)	15 (31.3%)		
	HTN	29 (25.9%)	3 (6.2%)		
	Others	29 (25.9%)	15 (31.3%)		
VA type	AVF	101 (90.2%)	39 (81.2%)	—	0.0310*
	AVG	4 (3.6%)	0 (0.0%)		
	TCC	7 (6.2%)	9 (18.8%)		
Age (year)	(m ± sd)	61.6 ± 11.0	64.0 ± 11.7	1.23	0.2188
Vintage (month)	median (IQR)	51.0 (25.0, 89.0)	59.0 (29.5, 90.0)	1.04	0.2971^#^
IDWG%	(m ± sd)	3.5 ± 1.3	3.9 ± 1.0	1.77	0.0800
uf (L)	(m ± sd)	2.3 ± 0.9	2.4 ± 0.7	0.72	0.4755
Pre-SBP (mmHg)	(m ± sd)	144.5 ± 14.9	133.5 ± 23.8	2.97	**0.0042**
Pre-DBP (mmHg)	(m ± sd)	80.3 ± 8.7	75.6 ± 12.1	2.46	**0.0165**
Kt/V	(m ± sd)	1.3 ± 0.3	1.4 ± 0.3	1.09	0.2777
BMI (Kg/m^2^)	(m ± sd)	21.9 ± 3.3	21.6 ± 2.8	0.51	0.6097
LDL-C (mmol/L)	(m ± sd)	2.5 ± 1.0	2.9 ± 0.9	0.22	**0.0276**

Abbreviations: ESRD = end-stage renal disease, VA = vascular access, AVF = arteriovenous fistula, AVG = arteriovenous graft, TCC = cuffed catheter, idwg = interdialysis weight gain, uf = average reality ultrafiltration volume, BMI = body mass index, LDL = low-density lipoprotein, Pre-SBP = predialysis Systolic Blood Pressure, Pre-DBP = predialysis Diastolic Blood Pressure, Kt/V: Urea clearance index

*Fisher’s exact test

^#^ Kruskal‒Wallis test was employed due to the skewed distribution

### Impact of frequent IDH on QoL

**Fig. 2:**
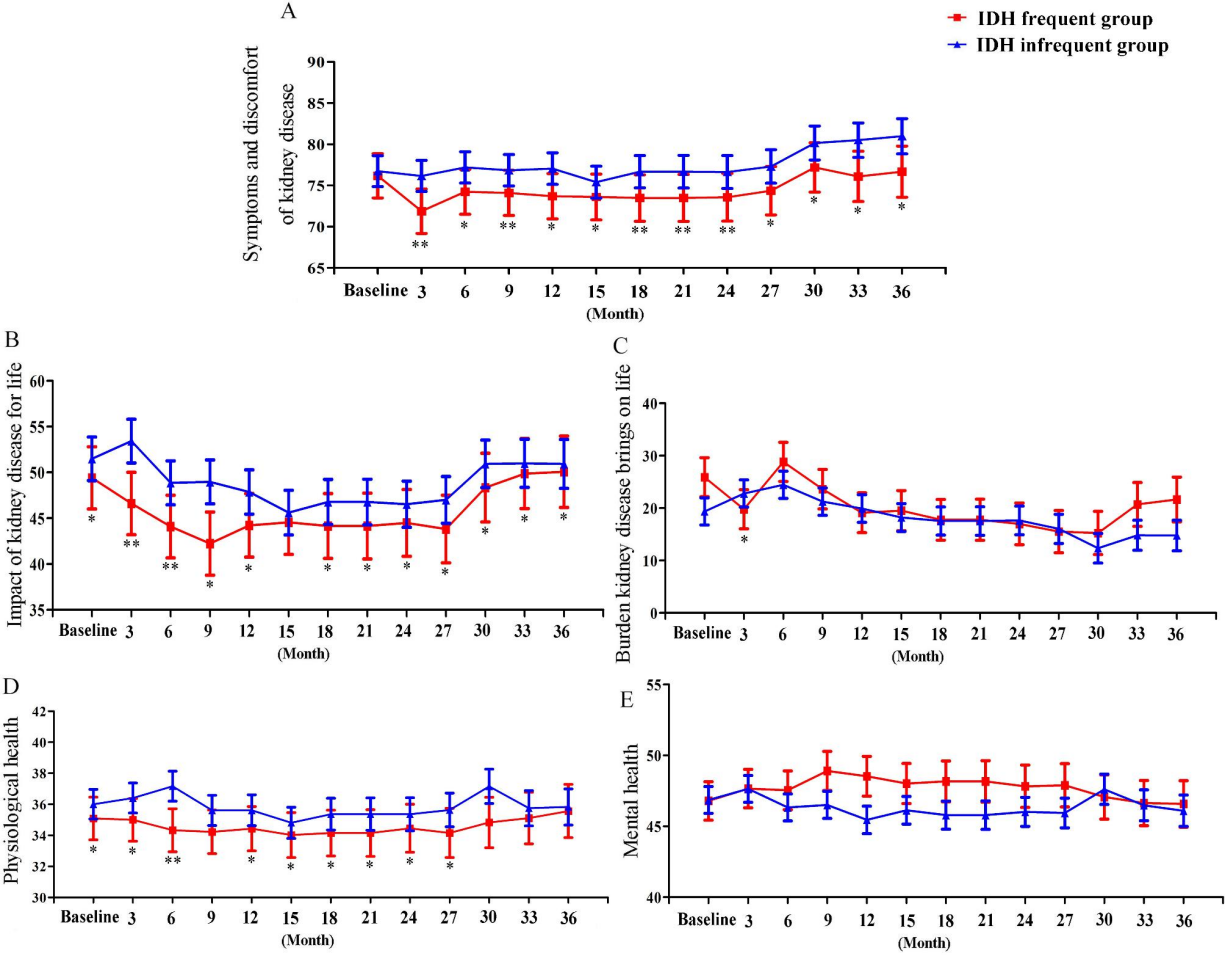
Impact of frequent IDH at baseline on all dimensions of QoL (**A:** Symptoms and discomfort of kidney disease; **B:** Impact of kidney disease for life; **C:** Burden kidney disease brings on life; **D:** Physiological health; **E:** Mental health),* *P* < 0.05 or ** *P* < 0.01

As shown in Table 2; Fig. 2, frequent or infrequent hypotension at baseline affected symptoms and discomfort of kidney disease or the impact of kidney disease at 36 months. The secondary outcomes showed that, those with frequent IDH had a statistically significant lower score of symptoms and discomfort of kidney disease dimension at all follow-up points (P < 0.05) compared to patients without frequent IDH at baseline. In terms of the impact of kidney disease on the life dimension, the QoL scores in the without frequent IDH group at baseline were higher than those in the frequent IDH group at all follow-up points (P < 0.05), except for month 15. For the physiological health dimension, patients with frequent IDH at baseline had lower scores than those without frequent IDH at baseline, and there was a statistically significant difference between the two groups at months 3, 6, and 12 to 27 (P < 0.05). Additionally, a significant between-group difference in the score of the burden kidney disease brings on the life dimension was observed only at month 3 (P < 0.05). However, there was no significant difference in the mental health dimension score between the two groups at any visit. Interestingly, the QoL scores of symptoms and discomfort of kidney disease and impact of kidney disease on life increased between months 27 and 30, regardless of whether patients had frequent hypotension at baseline.

**Table 2:** Influence of frequent IDH on QoL

		IDH frequent group	IDH infrequent group	Difference between groups	P value
Symptoms and discomfort of kidney disease	Baseline	74.05 (68.83, 79.27)	77.41 (73.75, 81.07)	-3.36 (-8.78, 2.06)	0.2240
	Month 3	69.27 (64.04, 74.51)	77.07 (73.40, 80.75)	-7.80 (-13.24, -2.36)	**0.0050**
	Month 6	71.49 (66.23, 76.75)	78.15 (74.47, 81.84)	-6.66 (-12.14, -1.19)	**0.0170**
	Month 9	70.58 (65.28, 75.87)	78.13 (74.43, 81.84)	-7.56 (-13.08, -2.04)	**0.0073**
	Month 12	71.45 (66.11, 76.78)	77.80 (74.06, 81.53)	-6.35 (-11.93, -0.77)	**0.0256**
	Month 15	70.01 (64.62, 75.40)	76.72 (72.95, 80.49)	-6.71 (-12.36, -1.06)	**0.0200**
	Month 18	69.97 (64.52, 75.42)	77.97 (74.16, 81.77)	-8.00 (-13.73, -2.26)	**0.0063**
	Month 21	69.97 (64.45, 75.49)	77.97 (74.12, 81.82)	-8.00 (-13.83, -2.16)	**0.0072**
	Month 24	69.75 (64.15, 75.35)	78.06 (74.16, 81.96)	-8.31 (-14.25, -2.37)	**0.0062**
	Month 27	70.84 (65.15, 76.53)	78.60 (74.65, 82.55)	-7.76 (-13.82, -1.70)	**0.0121**
	Month 30	73.79 (68.00, 79.57)	81.41 (77.40, 85.42)	-7.62 (-13.81, -1.43)	**0.0158**
	Month 33	73.79 (67.90, 79.68)	81.28 (77.20, 85.35)	-7.49 (-13.81, -1.17)	**0.0203**
	Month 36	74.14 (68.14, 80.13)	81.85 (77.71, 86.00)	-7.72 (-14.19, -1.25)	**0.0194**
Impact of kidney disease for life	Baseline	45.44 (38.87, 52.02)	52.96 (48.33, 57.58)	-7.51 (-14.31, -0.71)	**0.0305**
	Month 3	44.79 (38.20, 51.39)	54.02 (49.38, 58.65)	-9.22 (-16.05, -2.39)	**0.0082**
	Month 6	39.72 (33.09, 46.34)	50.53 (45.88, 55.18)	-10.81 (-17.68, -3.94)	**0.0021**
	Month 9	41.21 (34.55, 47.88)	49.22 (44.54, 53.89)	-8.00 (-14.93, -1.07)	**0.0236**
	Month 12	40.17 (33.45, 46.89)	49.38 (44.67, 54.10)	-9.21 (-16.22, -2.21)	**0.0100**
	Month 15	40.63 (33.84, 47.41)	47.07 (42.32, 51.82)	-6.44 (-13.54, 0.65)	0.0752
	Month 18	39.52 (32.66, 46.38)	48.55 (43.75, 53.35)	-9.03 (-16.23, -1.83)	**0.0140**
	Month 21	39.52 (32.57, 46.47)	48.55 (43.69, 53.40)	-9.03 (-16.35, -1.71)	**0.0157**
	Month 24	39.91 (32.86, 46.96)	48.27 (43.35, 53.18)	-8.36 (-15.81, -0.91)	**0.0280**
	Month 27	40.50 (33.34, 47.66)	48.21 (43.23, 53.19)	-7.71 (-15.31, -0.12)	**0.0466**
	Month 30	43.82 (36.54, 51.09)	52.65 (47.60, 57.70)	-8.83 (-16.59, -1.08)	**0.0256**
	Month 33	44.66 (37.26, 52.07)	52.98 (47.85, 58.11)	-8.32 (-16.24, -0.39)	**0.0396**
	Month 36	44.66 (37.13, 52.20)	53.01 (47.80, 58.22)	-8.35 (-16.45, -0.24)	**0.0435**
Burden kidney disease brings on life	Baseline	20.16 (12.88, 27.44)	21.41 (16.32, 26.50)	-1.25 (-8.88, 6.38)	0.7475
	Month 3	16.12 (8.82, 23.42)	24.03 (18.93, 29.14)	-7.91 (-15.57, -0.26)	**0.0428**
	Month 6	23.93 (16.60, 31.27)	26.15 (21.03, 31.28)	-2.22 (-9.92, 5.48)	0.5719
	Month 9	21.33 (13.95, 28.71)	21.86 (16.70, 27.01)	-0.53 (-8.29, 7.24)	0.8941
	Month 12	17.94 (10.51, 25.38)	20.07 (14.88, 25.26)	-2.13 (-9.97, 5.72)	0.5949
	Month 15	14.56 (7.05, 22.07)	19.96 (14.73, 25.19)	-5.40 (-13.34, 2.54)	0.1823
	Month 18	14.56 (6.97, 22.15)	18.56 (13.28, 23.85)	-4.01 (-12.06, 4.05)	0.3293
	Month 21	14.56 (6.87, 22.25)	18.56 (13.22, 23.91)	-4.01 (-12.18, 4.17)	0.3369
	Month 24	14.04 (6.24, 21.83)	18.56 (13.16, 23.97)	-4.53 (-12.85, 3.79)	0.2862
	Month 27	14.43 (6.52, 22.34)	16.16 (10.68, 21.65)	-1.74 (-10.21, 6.74)	0.6880
	Month 30	11.56 (3.53, 19.60)	13.54 (7.98, 19.10)	-1.98 (-10.62, 6.67)	0.6537
	Month 33	16.64 (8.47, 24.82)	16.16 (10.52, 21.81)	0.48 (-8.35, 9.31)	0.9155
	Month 36	17.29 (8.97, 25.62)	16.22 (10.49, 21.96)	1.07 (-7.95, 10.09)	0.8156
Physiological health	Baseline	33.62 (30.97, 36.28)	36.53 (34.67, 38.39)	-2.90 (-5.65, -0.16)	**0.0384**
	Month 3	33.42 (30.75, 36.08)	36.98 (35.11, 38.85)	-3.56 (-6.33, -0.79)	**0.0117**
	Month 6	33.67 (30.98, 36.36)	37.36 (35.48, 39.25)	-3.69 (-6.49, -0.89)	**0.0097**
	Month 9	33.32 (30.61, 36.04)	35.89 (33.99, 37.79)	-2.57 (-5.41, 0.27)	0.0764
	Month 12	32.60 (29.85, 35.36)	36.29 (34.36, 38.22)	-3.69 (-6.58, -0.79)	**0.0125**
	Month 15	32.15 (29.34, 34.95)	35.50 (33.55, 37.46)	-3.36 (-6.31, -0.40)	**0.0261**
	Month 18	32.45 (29.59, 35.31)	36.00 (34.01, 37.99)	-3.55 (-6.58, -0.52)	**0.0218**
	Month 21	32.45 (29.53, 35.37)	36.00 (33.97, 38.02)	-3.55 (-6.66, -0.43)	**0.0256**
	Month 24	32.75 (29.76, 35.74)	35.98 (33.91, 38.05)	-3.23 (-6.43, -0.03)	**0.0482**
	Month 27	32.68 (29.62, 35.75)	36.17 (34.05, 38.28)	-3.49 (-6.79, -0.18)	**0.0386**
	Month 30	34.05 (30.91, 37.20)	37.40 (35.23, 39.56)	-3.35 (-6.76, 0.06)	0.0544
	Month 33	34.33 (31.10, 37.56)	35.98 (33.76, 38.20)	-1.65 (-5.18, 1.87)	0.3570
	Month 36	34.40 (31.08, 37.72)	36.21 (33.94, 38.49)	-1.81 (-5.45, 1.82)	0.3282
Mental health	Baseline	45.26 (42.59, 47.93)	47.36 (45.49, 49.22)	-2.10 (-4.90, 0.70)	0.1423
	Month 3	46.34 (43.66, 49.02)	48.05 (46.18, 49.92)	-1.71 (-4.52, 1.11)	0.2338
	Month 6	45.95 (43.26, 48.65)	46.84 (44.96, 48.72)	-0.89 (-3.73, 1.95)	0.5389
	Month 9	46.81 (44.09, 49.53)	47.22 (45.32, 49.12)	-0.41 (-3.28, 2.47)	0.7819
	Month 12	47.27 (44.52, 50.02)	45.81 (43.89, 47.72)	1.46 (-1.45, 4.37)	0.3250
	Month 15	46.15 (43.36, 48.94)	46.75 (44.81, 48.69)	-0.60 (-3.56, 2.37)	0.6935
	Month 18	46.62 (43.78, 49.45)	46.28 (44.31, 48.24)	0.34 (-2.68, 3.36)	0.8245
	Month 21	46.62 (43.74, 49.50)	46.28 (44.28, 48.27)	0.34 (-2.75, 3.43)	0.8282
	Month 24	46.30 (43.37, 49.24)	46.50 (44.47, 48.53)	-0.20 (-3.36, 2.96)	0.9034
	Month 27	46.28 (43.28, 49.28)	46.45 (44.38, 48.52)	-0.17 (-3.41, 3.07)	0.9176
	Month 30	45.50 (42.44, 48.56)	48.13 (46.02, 50.24)	-2.63 (-5.95, 0.69)	0.1209
	Month 33	45.66 (42.53, 48.79)	46.74 (44.59, 48.89)	-1.08 (-4.50, 2.34)	0.5351
	Month 36	45.47 (42.27, 48.67)	46.41 (44.22, 48.61)	-0.94 (-4.45, 2.57)	0.5989

We explored the mean between-group difference in QoL scores over time and the variability of the between-group difference over time between frequent and infrequent IDH. The results showed that those with frequent IDH had a significantly poorer QoL regarding the dimensions of symptoms and discomfort of kidney disease and impact of kidney disease for life compared to patients with infrequent IDH. No significant interaction effects of the between-group difference and time were found. Details are shown in Table 3.

**Table 3:** Overall difference in QoL between patients with or without frequent IDH at baseline

	Overall test	F value	P value
Symptoms and discomfort of kidney disease	Group (With or without frequent IDH) ^a^	8.26	**0.0041**
	Group ×visit time interaction effect^b^	0.53	0.8942
Impact of kidney disease for life	Group (With or without frequent IDH) ^a^	7.19	**0.0074**
	Group ×visit time interaction effect^b^	0.30	0.9900
Burden kidney disease brings on life	Group (With or without frequent IDH) ^a^	0.61	0.4365
	Group ×visit time interaction effect^b^	0.94	0.5005
Physiological health	Group (With or without frequent IDH) ^a^	5.39	**0.0203**
	Group ×visit time interaction effect^b^	0.61	0.8364
Mental health	Group (With or without frequent IDH) ^a^	0.27	0.6019
	Group ×visit time interaction effect^b^	1.42	0.1474

a: Overall comparisons of mean QoL scores between the two groups; b: Tests for the interaction of the between-group difference and time

## Discussion

IDH occurs as a result of an interaction between the ultrafiltration rate, cardiac output, and arterial tone. In dialysis clinics, the presence of IDH seriously affects dialysis treatment and the adequacy of dialysis, leading to damage to vital organs, impaired quality of life (QoL) and increased all-cause mortality [4, 10, 11]. To better understand the effect of IDH on patient QoL, this study investigated the association between frequent IDH—defined as a predialysis BP and nadir SBP below predefined thresholds while frequency no less than 30% of hemodialysis sessions—and QoL dimensions [4]. The results showed that frequent IDH was significantly associated with poorer QoL in two dimensions: symptoms and discomfort of kidney disease and impact of kidney disease for life.

It has long been generally accepted that hemodialysis treatment may reduce patients’ QoL. However, studies exploring the correlation between IDH and QoL are very limited and mainly focus on the uncomfortable symptoms caused by hemodialysis. One study found that the EBPG definition of IDH does not capture aspects of intradialytic symptomatology that are relevant for patient QoL, although there is a significant association between QoL and a simple patient-reported intradialytic symptom score [7]. Our study found a significant association between frequent IDH and three dimensions of the KDQOL™-36 scale. This difference may be attributed to the fact that the definition of IDH adopted in our study did not simply consider the decrease or absolute value of BP as the definition for IDH but examined the nadir SBP threshold and predialysis BP, including both absolute and relative decreasing amplitude. This is more consistent with the pathophysiological differences of different BP and volume changes in patients with diverse basic BP The potential advantage of using the nadir SBP threshold based on certain restrictions on predialysis BP as comprehensive diagnostic criteria for IDH is its ability to identify patients with IDH who experience slow or sharp BP drops to a certain extent. Additionally, the definition of IDH used in our study also took into account the plummeting of SBP in patients with high predialysis BP, namely, those below 100 mmHg with predialysis BPof ≥ 160 mmHg. This criterion, derived from a large population retrospective study, increases the nadir SBP for those with high predialysis BP. At the same time, this definition reduces the possibility of bias due to subjective and individual differences compared with those definitions based on symptoms and clinical interventions [3, 12].

Clinical symptoms are the most intuitive indicators for determining the presence of IDH. The KDQOL™-36 scale measures two dimensions of clinical symptoms, namely, symptoms and discomfort and physical health, and an extensive dimension of the impact of kidney disease for life [13]. Pathophysiological studies have shown that reduced effective arterial blood volume leads to cardiac insufficiency and reduced cardiac output when the rate of fluid removal surpasses the rate of plasma refilling during dialysis; subsequently, cardiovascular and neurohormonal compensatory responses occur during dialysis, eventually leading to the emergence of significant IDH [14–16]. Hemodialysis can also lead to ischemic events in multiple organs, such as the heart, intestines, brain, and kidneys [17–21]. Organ ischemia or hemodynamic instability can manifest as nausea, dizziness, or cramps. A survey of 550 patients on maintenance hemodialysis found that 74% experienced cramps, 63% experienced dizziness, and 54% experienced headaches [22]. In contrast, some studies have reported a low incidence of discomfort symptoms. A prospective cohort study of 124 hemodialysis patients found that only 21.4% developed clinical symptoms, including cramping, dizziness, and nausea, at 8.8%, 4.9%, and 2.6%, respectively [23]. A questionnaire survey of 77 hemodialysis patients found that dizziness and cramps were strongly associated with changes in SBP. The percentages of patients with nausea, dizziness, and cramps were 22.1%, 12.3%, and 7.5%, respectively [24]. These findings show that there is a significant degree of variability in reported symptoms during hemodialysis, which highlights the importance of assessing patients’ symptoms over a specific period of time (in our study, this was three months). The IDH definition in our study enabled us to better screen patients with IDH, thereby revealing the characteristics of clinical symptoms and reduced QoL in these patients. Even if the frequency of IDH decreases during long-term treatment and follow-up, symptoms present at baseline may still have some impact on QoL. This impact is mainly reflected in the reduction in QoL during the follow-up period. In a clinical study of 1846 hemodialysis patients, SBP decline (from the predialysis value to the dialysis session nadir, per 10-mm Hg decrease) was associated with greater risk for muscle cramping, headache, chest pain, vomiting, and lightheadedness [5]. The totality of symptoms, including their effect on patients’ health-related quality of life such as muscle spasticity, headache, chest pain, vomiting and dizziness and the ability to participate in life, is described as the ‘symptom burden’. Kalantar-Zadeh et al. believe that symptom burden will adversely affect the quality of life of hemodialysis patients, and patient-centered symptom burden management methods should be developed [25]. Furthermore, previous studies have shown that hemodialysis patients are affected by nutritional and inflammatory conditions, and IDH patients often have the characteristics of sarcopenia such as low skeletal muscle mass and low muscle strength [26]. Patients with low skeletal muscle mass and low muscle strength may have poor quality of life due to lack of physical activity [27]. This may be one reason for the lower score of the KDQOL™-36 scale in patients with frequent IDH. Combined with the relationship between frequent IDH and all-cause mortality [4], our study findings that frequent IDH, with the IDH definition used in our study, was significantly associated with symptoms and discomfort of kidney disease and the impact of kidney disease for life are further explained. In addition, we retrospectively compared the QoL score of the KDQOL™-36 scale between the IDH frequent group and the infrequent group. The advantage is that the KDQOL™-36 scale is blind to the objective of the study. However, in a prospective study, the patient report could be biased by the mandatory information about the study objectives.

The QoL scores of symptoms and discomfort of kidney disease and impact of kidney disease on life increased between months 27 and 30, regardless of whether patients had frequent hypotension at baseline. We switched from paper to electronic questionnaires between months 27 and 30. Previous studies have shown that changes in QOL scores during follow-up may be due to many factors other than the intervention, such as the effect of subject attention, and survey burden [28]. However, it is not certain whether the increase in scores during this period is related to the change in the form of the questionnaire at present. Furthermore, it was not possible to judge whether the increased score was within the range of minimal clinically important difference for the KDQOL™-36 scale. According to our search, at present, there is no relevant literature measuring the minimal clinically important difference for the kidney disease impact on life by using the KDQOL™-36 scale in hemodialysis patients, and the differences in the minimal clinically important difference in the SF-12 subscale of the KDQOL™-36 in the nondialysis population are inconsistent [29, 30]. Further studies are needed in the future to understand the underlying mechanisms of IDH-related symptoms and to provide optimal dialysis treatment for patients. In clinical practice, IDH has been postulated to reduce QoL, but robust evidence has been lacking. One potential explanation for this may be the lack of uniform diagnostic criteria for IDH. In 2005, K/DOQI issued the first official definition of IDH, which stipulates that a 20 mmHg drop in SBP or a 10 mmHg drop in mean arterial pressure during or after dialysis in combination with clinical events and interventions should be considered IDH [3]. Unfortunately, this definition did not include grading recommendations due to the lack of evidence in the guidelines and has remained unchanged for years, indicating its lack of evidence-based medical support. This absence of uniform or evidence-based diagnostic criteria for IDH impedes the progress of IDH prevention and treatment. In the present study, we found an association between frequent IDH and QoL under the definition of an absolute nadir SBP < 90 mmHg when predialysis SBP < 159 mmHg (or < 100 mmHg when predialysis BP ≥ 160 mmHg). This definition might provide clinicians with a reference for selecting appropriate diagnostic criteria for IDH during follow-up and scientific research. Moreover, it has some practical advantages in data collection due to its simple parameter attribute [3, 18]. Given the findings of the present study, the definition is also associated with QoL dimensions of subjective symptoms and the impact of kidney disease, which may give it greater application value and a wider range of application scenarios.

This study has some limitations. First, as an observational study, there may be confounding factors leading to confounding bias. To provide more accurate effect estimates, we adjusted for potential confounders such as age, etiology of ESRD, VA type, and dialysis duration in the comparative analysis. However, owing to the small sample size, not all potential confounders could be taken into consideration. Second, the design of a single-center retrospective study with a limited sample size may introduce selection bias. Third, we used the KDQOL™-36 scale in this study, which lacks an investigation of subjects’ recovery time from fatigue.

The findings of the study suggest an association between frequent IDH and QoL dimensions of symptoms and discomfort of kidney disease and the impact of kidney disease on life dimension under the definition of frequent IDH whereby an absolute nadir SBP < 90 mmHg occurs no less than 30% of hemodialysis sessions when predialysis SBP < 159 mmHg (or < 100 mmHg when predialysis BP ≥ 160 mmHg). In the future, large-scale, multicenter prospective studies are needed to further investigate the diagnostic criteria for IDH in patients on maintenance hemodialysis and their clinical application value.

## Data Availability

The datasets used and/or analyzed during the current study are available from the corresponding author on reasonable request.
